# Exploring the cause of initially reactive bovine brains on rapid tests for BSE

**DOI:** 10.1080/19336896.2015.1115945

**Published:** 2015-12-21

**Authors:** Sandor Dudas, Jace James, Renee Anderson, Stefanie Czub

**Affiliations:** Canadian Food Inspection Agency; National Center for Animal Disease; Lethbridge, AB, Canada

**Keywords:** bovine spongiform encephalopathy;, rapid testing,, confirmatory testing;, diagnostics;, bacterial contamination;, false positive

## Abstract

Bovine spongiform encephalopathy (BSE) is an invariably fatal prion disease of cattle. The identification of the zoonotic potential of BSE prompted safety officials to initiate surveillance testing for this disease. In Canada, BSE surveillance is primarily focused on high risk cattle including animals which are dead, down and unable to rise, diseased or distressed. This targeted surveillance results in the submission of brain samples with a wide range of tissue autolysis and associated contaminants. These contaminants have the potential to interfere with important steps of surveillance tests resulting in initially positive test results requiring additional testing to confirm the disease status of the animal.

The current tests used for BSE screening in Canada utilize the relative protease resistance of the prion protein gained when it misfolds from PrP^C^ to PrP^Sc^ as part of the disease process. Proteinase K completely digests PrP^C^ in normal brains, but leaves most of the PrP^Sc^ in BSE positive brains intact which is detected using anti-prion antibodies. These tests are highly reliable but occasionally give rise to initially reactive/false positive results. Test results for these reactive samples were close to the positive/negative cut-off on a sub set of test platforms. This is in contrast to all of the previous Canadian positive samples whose numeric values on these same test platforms were 10 to 100 fold greater than the test positive/negative cut-off. Here we explore the potential reason why a sample is repeatedly positive on a sub-set of rapid surveillance tests, but negative on other test platforms.

In order to better understand and identify what might cause these initial reactions, we have conducted a variety of rapid and confirmatory assays as well as bacterial isolation and identification on BSE positive, negative and initially reactive samples. We observed high levels of viable bacterial contamination in initially reactive samples suggesting that the reactivity may be related to bacterial factors. Several bacteria isolated from the initially reactive samples have characteristics of biofilm forming bacteria and this extracellular matrix might play a role in preventing complete digestion of PrP^C^ in these samples.

## ABBREVIATIONS

BSE: bovine spongiform encephalopathy
PrP^C^: cellular prion protein
PrP^Sc^: misfolded/disease associated prion protein
TSE: transmissible spongiform encephalopathy
PK: proteinase K
PrP^Sc^(sen): misfolded/disease associated prion protein sensitive to protease digestion
PrP^Sc^(res): misfolded/disease associated prion protein resistant to protease digestion
IR: initially reactive
ICA: immuno-chromatographic assay
PK EIA: proteinase K based enzyme immuno-assay
LIG EIA: affinity ligand based immuno-assay
WB: standard western blot
SAF-WB: scrapie associated fibril Immuno-Blot
IHC: immunohistochemistry
BA: blood agar
NA: nutrient agar
BHIA: brain heart infusion agar
BHIB: brain heart infusion broth
BLAST: basic local alignment search tool

## INTRODUCTION

Bovine spongiform encephalopathy (BSE) is an invariably fatal prion disease of cattle.^1^ BSE surveillance and monitoring became an international focus following the discovery that the consumption of BSE contaminated beef products caused a similar fatal neurodegenerative disease in humans (variant Creutzfeldt-Jakob Disease).^2^ In Canada, the surveillance program for BSE in cattle is targeted at 4 specific categories of animals over 30 months of age. These categories include animals which are diseased, distressed, down/unable to rise or dead (the “4 D's”).^3^ With a focus on these risk groups, brainstem samples collected on farm are often autolyzed and contaminated with bacteria and other debris including hair, soil and feces. Tissue condition can additionally be influenced by inter-current diseases or absorbed/ ingested toxins or medications. These factors affect the tissue composition/sample quality and have the potential to impact diagnostic test performance and the results generated.

Detection of a protease resistant, misfolded, aggregating form of the normal host encoded prion protein is the main criteria used to identify animals and humans infected by prion diseases.^4^ It is also widely accepted that this misfolded prion protein (PrP^Sc^), devoid of any detectable genetic material, is the infectious entity capable of transmitting prion diseases.^5^ Diagnosing transmissible spongiform encephalopathies (TSE) is complicated by this unique protein only infectious agent. Most rapid tests used to detect prion pathogens in animals exploit unique characteristics of PrP^Sc^, including its increased resistance to proteinase K (PK) digestion. In these platforms, samples are subjected to PK digestion which degrades the normal PrP^C^ and a portion of protease sensitive misfolded prions (PrP^Sc^(sen)) but leaves the globular domain of aggregation prone, protease resistant prion largely unaffected (PrP^Sc^(res)). Anti-prion antibodies are then used to detect the intact PrP^Sc^(res). BSE samples submitted for surveillance in Canada are tested using proteinase K based rapid tests. While these test platforms are extremely robust and sensitive, they occasionally generate initially reactive results requiring further testing for confirmation of the sample disease status.

Misfolding and aggregation of PrP^C^ into PrP^Sc^ not only changes the relative protease resistance of the protein but it also changes its binding properties. Identification of ligands which specifically bind PrP^Sc^ is an alternative strategy used for prion disease surveillance.^6^ In this detection system the PrP^Sc^ specific ligand is immobilized in the well of a plate and test samples are allowed to incubate in the wells. If PrP^Sc^ is present, it binds to the ligand in the well and is detected using prion protein specific antibodies. The ligand based test provides an advantage to tests relying on PK digestions because it has the potential to bind and detect both PrP^Sc^(res) and PrP^Sc^(sen) which could explain the increased test sensitivity when compared to several of the PK based rapid tests.^7,8^ From the start of 2011 to the end of 2014, the Canadian BSE surveillance network has tested over 130,000 samples. Of these samples, 10 were submitted for confirmation (tested above the positive/negative cut-off on initial and repeat testing on a single test platform) and 2 were confirmed as BSE positive. That leaves approximately 0.006% of samples, or 8 out of 130,000, that fall into the category of reactive/false positive.

With Canadian BSE surveillance testing relying on the proteolytic digestion of PrP^C^ and subsequent immuno-detection of PrP^Sc^(res), contaminants that alter protease digestion efficiency could lead to confounding/reactive results. Studies exploring the effects of tissue quality and bacterial contamination on the generation of divergent test results have demonstrated that misfolded prions are not quickly degraded by autolysis and positive samples are accurately diagnosed even after severe decomposition.^9^ Of additional concern are potential contaminants inhibiting protease activity or protecting cellular prion proteins resulting in incomplete PK digestion and a reactive rapid test result. If reactive twice on a single rapid test platform, Canadian BSE testing guidelines indicate that the sample requires additional testing to confirm its disease status. When conflicting results are generated between test replicates or different test platforms, the question looms whether these samples truly harbor disease associated, protease resistant, transmissible prions or not. Multiple laboratories have reported this occurrence with different theories to explain the results.^10,11^

To better understand “initially reactive” (IR) samples detected in Canada, surveillance samples which were initially reactive with one or more rapid test were further evaluated. Testing these samples using confirmatory molecular methods indicates the absence of known types of PrP^Sc^ or PrP^Sc^(res). On standard western blot, the molecular weight of the largest immuno-reactive protein detected in the IR samples is smaller (˜26 kDa) than the characteristic di-glycosylated PrP^Sc^(res) (˜29 kDa) and binds preferably to antibodies detecting the C-terminal part of the protease resistant PrP core. This suggests preservation of the C-terminal globular domain of PrP^C^ as a result of incomplete denaturation/digestion. All IR samples included in this study generated similar western blot profiles and, as with other studies reporting IR samples, all of them were autolysed.^10,11^ It is conceivable that high levels of an autolysis-associated microbial population may be the underlying cause. Bacteria isolated from IR and BSE negative samples were sequenced to identify the types present in the different samples. The bacteria isolated from the IR samples often clumped together and/or adhered to the broth culture vessel wall which is indicative of biofilm forming bacteria. Biofilms are known to dramatically increase bacterial resistance to chemotherapy and may serve to inadvertently prevent PrP^C^ denaturation and/or degradation resulting in a signal on rapid tests for BSE.^12^

## MATERIALS AND METHODS

### Samples

Samples from the Canadian BSE surveillance program were grouped into 2 categories; initially reactive (IR) and BSE negative. BSE negative samples were defined as surveillance samples which gave a result of “BSE not detected” based on the kit defined positive/negative cut-off or defined test criteria. Initially reactive samples were BSE surveillance samples that were consistently above the kit defined positive/negative cut-off value on one or more of the rapid screening tests. A total of 10 BSE negative samples were included in this study, 5 were in fair condition (N1 to N5, **Table 1**)(still solid tissue with identifiable landmarks) and 5 were in poor condition (N6 to N10, **Table 1**; autolysed, liquid, landmarks gone or difficult to identify). Four initially reactive samples had tissue available for use in this study; 2 were in fair condition (R1 and R2, **Table 1**) and 2 were in poor condition (R3 and R4, **Table 1**). Two fair (P1 and P2, **Table 1**) and 2 poor quality (P3 and P4, **Table 1**) BSE positive tissue samples from Canadian classical BSE field cases were used for positive controls (**Table 1**).

**TABLE 1.** Sample information and BSE rapid and confirmatory test results

Sample ID	Tissue Quality	ICA^a^	WB^b^	PK EIA^c^	Lig EIA^d^	SAF-WB^e^	IHC^f^
R1	Fair	140.8(+)	+/-	0.018(-)	0.041(-)	-	-
R2	Fair	98.5(+/−)	–	0.056(-)	0.033(-)	+/-	+/-
R3	Poor	104.25(+)	+/-	0.022(-)	0.042(-)	+/-	-
R4	Poor	125.5(+)	+/-	0.014(-)	0.041(-)	+/-	-
N1-N5	Fair	0(−)	NT	NT	NT	NT	NT
N6-N10	Poor	0(−)	NT	NT	NT	NT	NT
P1	Fair	2406(+)	+	1.542(+)	1.853(+)	+	+
P2	Fair	7028(+)	+	3.497(+)	3.902(+)	+	+
P3	Poor	6558(+)	+	3.269(+)	3.876(+)	+	+
P4	Poor	428(+)	+	0.726(+)	1.024(+)	+	+

Four initially reactive (R1-R4), 10 BSE negative (N1-N10) and 4 BSE positive (P1-P4) tissue samples evaluated. Each of the 3 groups of samples had an equal number of fair and poor quality tissues included.

a Immuno-Chromatographic Assay, Negative to positive cut off ˜80-100 optical density units (ODU).

b Western Blot, - no signal, +/− signal of unexpected size/pattern, + expected positive signal.

c Proteinase K purification based Enzyme Immuno-Assay, Negative to Positive cut off ˜0.200 ODU.

d Affinity ligand purification based Enzyme Immuno-Assay, Negative to Positive cut off ˜0.200 ODU.

e Scrapie Associated Fibril Western Blot, same as western blot.

f Immunohistochemistry, - no specific labeling, +/− labeling in an unexpected pattern, + expected positive labeling.

### Molecular Detection of PrP^Sc^

All tissue samples used were tested on an immuno-chromatographic assay (ICA)(Prionics, 30000); a proteinase K based enzyme immuno-assay (PK EIA)(BioRad, 355-1144/355-1194); an affinity ligand based immuno-assay (LIG EIA)(IDEXX, 99-08600); a standard western blot (WB)(Prionics, 12000); the Scrapie Associated Fibril Immuno-Blot (SAF-WB) and immunohistochemistry (IHC). These tests are routinely used at the Canadian National and OIE Reference Laboratory for BSE and testing was performed by certified analysts. Testing with commercial kits was performed following the manufacturer's instructions (ICA, PK EIA, LIG EIA, WB). For results generated with non-commercial BSE tests, protocols controlled by the CFIA Lethbridge Laboratory ISO 17025 Quality Assurance (QA) office were followed (SAF-WB and IHC).

The ICA, PK EIA and WB all required tissue homogenization followed by proteinase K digestion. Digested samples were subjected to a number of protocol specific steps to allow for immuno-detection of PK resistant PrP^Sc^. Each kit has defined criteria for results interpretation to identify samples as positive or negative. The LIG EIA does not use a proteinase K digestion step. Brain tissue samples are homogenized and conditioned via several steps so PrP^Sc^ in a sample will bind to the immobilized PrP^Sc^-specific ligand. The bound protein is detected using a prion protein specific immuno-assay and results are defined by cut off values calculated based on optical densities of the kit supplied controls. All of the rapid surveillance tests use a fixed amount of sample tissue homogenate and have no steps to selectively concentrate or purify PrP^Sc^. The SAF Immuno-blot requires a much larger tissue input and the PrP^Sc^ is purified from this tissue amount, resulting in close to a 3 log increase in sensitivity for the known BSE types compared to the standard western blot.

The SAF Immuno-blot protocol was adapted from Hilmert and Diringer.^13^ Briefly, brain stem samples were homogenized in brain lysis buffer. The homogenate was centrifuged to remove larger cellular debris; the supernatant was transferred to new ultracentrifuge tubes and spun. The supernatant was discarded and the pellet was thoroughly re-suspended in TRIS buffer and proteinase K digested. Digested samples were carefully pipetted on top of a 20% sucrose cushion in a new tube and ultra-centrifuged. The supernatants and sucrose cushions were discarded and the pellets were re-suspended in SDS PAGE sample buffer, sonicated, boiled, separated on an SDS PAGE gel and transferred onto a PVDF membrane for immuno-detection. Positive samples were identified by immuno-reactivity, molecular weight analysis and by the expected glyco-form ratios.

### Immunohistochemistry for PrP^Sc^

Tissue from IR and BSE negative samples were fixed in 10% neutral buffered formalin, processed, embedded in paraffin blocks and sections were cut at 3-5 µm. Slide mounted tissues were autoclaved in a target retrieval solution (Dako, S169984-2), blocked in 2% normal goat serum and incubated overnight at 4°C with primary antibody 6H4 (Prionics, 01-010). The Envision + HRP conjugated polymer kit (Dako, K400611-2) was used for colorimetric detection of bound primary antibody. Slides were evaluated for the presence of immuno-labeling characteristic of prion infection using light microscopy.

### Growth and Isolation of Contaminating Bacteria

IR and BSE negative tissue samples were trimmed and placed in sterile screw cap tubes. Sterile water was added to make a 20% w/v homogenate and 4 × 3 mm sterile ceramic beads were added to each tube. The tissue, beads and water were homogenized by vigorous shaking in the BioRad Precess 48 (BioRad, 359-0200) for 2 cycles of 30s. Homogenates were diluted in sterile water and were plated on Blood Agar (BA), Nutrient Agar (NA) and Brain Heart Infusion Agar (BHIA). The plates were incubated overnight at 37°C and bacterial colonies were enumerated and further characterized.

Bacterial colonies with unique gross morphologies from each sample were grown in 3 mL of Brain Heart Infusion broth (BHIB) for approximately 16 hours in a shaking 37°C incubator. BHIB culture was centrifuged at 13,000 rpm for 3 minutes to pellet the bacterial cells. Nucleic acid extraction was performed on cell pellets using the Qiagen DNeasy Blood and Tissue Kit (Qiagen, 69504). The extracted DNA served as the template for 16s ribosomal RNA PCR reactions.

### PCR and Sequencing

Primers specific for bacterial 16s ribosomal RNA were used to generate PCR products for sequencing (Forward Primer 5'-AGA GTT TGA TCC TGG CTC AG-3′, Reverse Primer 5′-GGT TAC CTT GTT ACG ACT T-3′).^14^ The PCR reaction mix contained 100 mM KCl, 100 mM Tris (pH 8.3), 100 mM MgCl, 0.01% w/v gelatin, 100 µM forward primer, 100 µM reverse primer, and 5 U/µL Invitrogen Platinum Taq DNA polymerase (Life Technologies, 10966018). Fifty microliter PCR reactions (48 µL reaction mix + 2 µL extracted DNA) were held at 94°C for 10 min, then cycled 30 times through 94°C for 1 min, 50°C for 1 min, and 72°C for 2 min. A final extension incubation of 72°C for 10 min completed the amplification.

To purify specific PCR products for sequencing, reactions were separated on a 1% agarose-TAE gel containing ethidium bromide. After separation, bands were excised and the PCR products were purified using the Qiagen Qiaquick Gel Extraction Kit (Qiagen, 28704). PCR products were sent for sequencing (Eurofins MWG Operon Inc.) and the raw sequence data was processed using Sequencer (Version 5.0, Gene Codes). Aligned consensus sequences created from at least one sequence strand in each direction were entered and searched using the Basic Local Alignment Search Tool (BLAST) against the PubMed database to identify the bacterial strains with the highest sequence homology to the isolated bacteria.

Bacteria identified in both IR and BSE negative samples were removed as potential candidates for causing interference in the diagnostic tests. The bacterial isolates only found in the IR brains were cultured in brain heart infusion broth for ∼16 hours at 37°C and the number of CFU/mL was determined by culturing a dilution of the broth on blood agar plates. Immediately after plating the broth for enumeration, another aliquot was removed and spiked into a 20% sterile BSE negative brain homogenates in water. This was incubated for 24 hours at 37°C after which an aliquot was removed to determine the growth/viability of the bacteria in brain tissue homogenate. The remaining homogenate was diluted to 10% (w/v) in homogenization buffer, homogenized and tested on the ICA and standard WB rapid surveillance tests to determine if a specific bacterial contamination and/or growth would result in consistent initially reactive results.

Bacteria isolated from the IR samples and BSE rapid test negative tissue samples with a similar level of autolysis were tested using a published biofilm assay.^15^ Briefly, bacteria were grown in brain heart infusion broth overnight at 37°C. Bacterial broth was diluted 1:100 in biofilm assay media (22 mM KH_2_PO_4_, 40 mM K_2_HPO_4_, 15 mM (NH_4_)_2_SO_4_, 1 mM MgSO_4_, 0.4% w/v arginine). The samples were incubated on a poly-vinyl 96 well plate at 37°C for 24 hours, media was then removed and the wells were washed with water. Crystal violet solution (0.1%) was added to each well and the plates were incubated for 15 min at room temperature. The crystal violet solution was discarded and the plates were washed 3 times with water and allowed to dry. Glacial acetic acid was added to each well (30% v/v) to solubilize the crystal violet bound to the biofilm formed in the plate wells. The glacial acetic acid/crystal violet was transferred to a new flat bottom 96 well plate and the absorbance was read at 490 nm. The absorbance results for at least 4 replicates for each bacterial isolate were averaged to quantify the amount of crystal violet and in turn the amount of biofilm growing for the different bacterial isolates.

### Gram Staining of Tissue Sections

Brain sections cut from paraffin tissue blocks were Gram stained following the Hucker-Conn Gram Staining protocol with minor modifications.^16^ Slide mounted tissue sections were flooded with crystal violet solution for 1 min, rinsed with water and flooded for 1 min with Grams Iodine solution. Iodine solution was washed away with water and the tissues were decolourized in a gentle stream of 100% acetone. Slides were rinsed with water, counter stained in neutral red, cover slipped and visualized using light microscopy.

## RESULTS

### Detection of Disease Associated Prions

**FIGURE 1.**
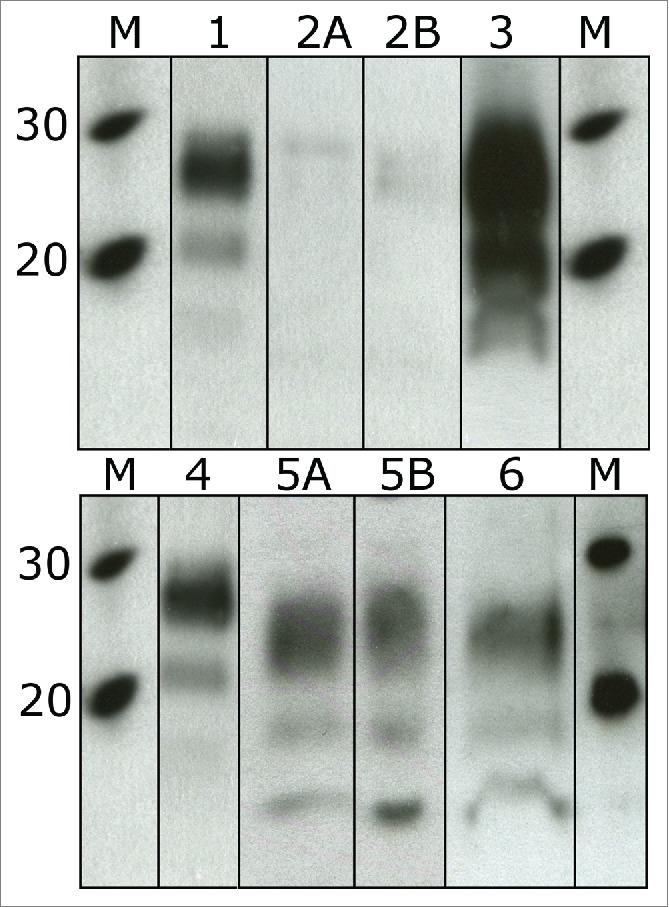
Western blots using the anti-PrP anitbody 6H4. Sample 1 and 4 are a mid-range positive BSE sample. 2A and B are replicate lanes of a poor tissue quality weak BSE positive sample (P4). Lane 3 is the same weak BSE positive sample (P4) after SAF purification. 5A and B are replicate lanes of a poor tissue quality initially reactive sample (R4) and lane 6 is the same sample after SAF purification. The increase in immuno-reactivity is obvious when comparing lanes 2A and B to lane 3.There is no obvious increase when comparing lanes 5A and B to lane 6.

Testing of IR samples generated conflicting results on the rapid surveillance tests. Initially reactive results were generated with some tests utilizing proteinase K digestions but not with some of the other PK based or the ligand affinity based rapid test. Using the affinity ligand based test and the PK EIA test, no reactivity in any of the BSE negative or initially reactive samples was detected (**Table 1**). The standard WB uses PK to isolate PrP^Sc^(res) from the omni-present PrP^C^ and results for the IR samples show immuno-reactivity on this platform. The blots had a banding pattern similar to the glyco-profile of PrP^Sc^(res) but with a notably lower molecular weight than any previously described BSE type. The di-glycosylated band of a classical BSE positive sample is approximately 3 kDa larger than the corresponding band in the initially reactive samples (**Fig. 1**). Based on the western blot reactivity of both core (6H4, Prionics AG) and C-terminal antibodies (94B4, J. Langeveld) combined with limited reactivity with an N-terminal/core antibody (P4, Roche), PK seems to cleave additional amino acids predominantly from the less structured N-terminal portion of the prion protein leaving most of the core and C-terminus intact. (Results not shown.)

**FIGURE 2.**
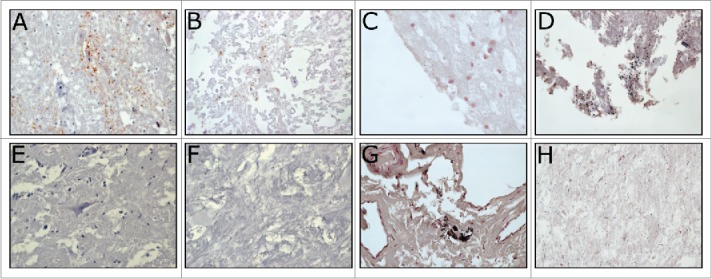
Immunohistochemistry (IHC) and Gram stains of confirmed BSE positive and initially reactive surveillance brain tissue samples. Tissue sections were immuno-stained with anti-prion antibodies (6H4 or L42) or Gram stained to identify the presence of Gram positive and/or negative bacteria. The BSE positive IHC images (**A, B**) show the coarse, granular labeling of PrP^Sc^ in both fair (**A**) and poor quality (**B**) tissue samples. The initially reactive samples (**E, F**) do not have this staining pattern in IHC. Gram positive and Gram negative bacteria can be seen in the BSE + and initially reactive tissues of fair (**C, G**) and poor quality (**D, H**). In fair quality tissues, bacteria is usually of a single type and is limited to the tissue peripheries (**C, G**). Poor quality tissues have an abundant and diverse bacterial flora distributed throughout the sections (**D, H**).

**FIGURE 3.**
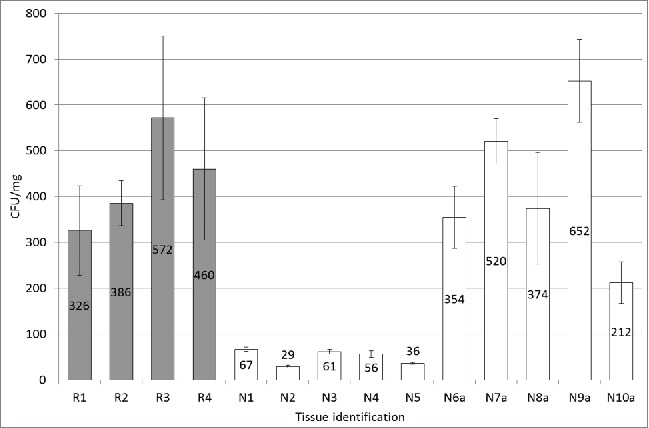
The viable aerobic bacterial populations cultured from surveillance brain tissue samples. A non-reactive sample (N9a) had the highest individual colony count. The average CFU/mg of tissue for the initially reactive samples was 436 (N1 to N5) while the autolysed non-reactive samples averaged 422 CFU/mg of tissue (N6a to N10a).

The lack of reactivity of the IR samples on the affinity ligand based test and the molecular weight shift of reactive bands on the standard western blot indicates the PrP causing the reactivity seen on the PK based test platform has different molecular characteristics and folded structure then previously characterized PrP^Sc^. A similar reactivity is seen when known negative brain samples are not homogenized sufficiently in the homogenization solution provided in the test kits. This was discovered when known negative brain homogenates were inoculated with individual bacterial isolates in an attempt to recreate the initially reactive samples. For these tests, 20% brain in water was inoculated with bacteria and incubated at 37°C for 24 h before being mixed with an equal volume of 2x homogenization buffer. If these samples were not re-homogenized (in tubes with ceramic beads) before testing, elevated values were seen on the ICA and standard WB profiles were similar to those of the IR samples. This reactivity was eliminated by bead homogenizing the samples after homogenization buffer was added.

### SAF and IHC

The World Organization for Animal Health (OIE) recognizes immunohistochemistry (IHC) and the scrapie associated fibril (SAF) immuno-blot as the gold standard tests for BSE. These methods are also used to confirm the diagnosis of bovine brain samples with discordant results on multiple rapid tests.^17^ Initially reactive as well as BSE negative and positive samples were tested using both of these methods. Results generated with the initially reactive samples were not indicative of a weak, previously characterized BSE positive sample. Using IHC, the IR samples sometimes had diffuse non-specific or background staining. None of the prion specific antibodies used exhibited the distinct granular staining reaction characteristic of BSE, even evident when BSE positive tissue was very autolysed (**Fig. 2**).

Samples were tested on the SAF immuno-blot to selectively concentrate PrP^Sc^ from a large amount of tissue. We have previously shown that this test is at least 10–100 fold more sensitive than the most sensitive rapid test (LIG EIA)^7,8^ and a sample with rapid test results slightly above or even elevated but below the test defined positive/negative cut-off should be easily detected. Four grams of brain tissue was processed and concentrated into a single sample, half of which was used for western blot detection (2 g tissue equivalent). In very weak BSE positive samples, which are difficult to diagnose on standard tests, the SAF purification step significantly increases the concentration of PrP^Sc^ resulting in a much more intense western blot signal. However, when the IR samples were tested using the SAF immuno-blot; there was no evidence that PrPSc had been concentrated as there was no significant increase in blot signal intensity (**Fig. 1**). While the blot results generated were not completely clean for some of the initially reactive samples, as is seen with good quality negative tissue samples, a similar level of reactivity was present on the SAF immuno-blots (2000 mg tissue equivalent on the SAF-WB) as on the rapid surveillance western blot (0.43 mg tissue equivalent on the WB). Estimates suggest that the SAF purification is around 20% efficient at purifying PrP^Sc^ from brain tissue.^11,18^ Assuming our SAF purifications efficiency is comparable, we expect approximately a 900 fold increase in the signal intensity from the surveillance western blot to the SAF immuno-blot (WB 0.43 mg vs SAF-WB 400 mg). With no notable increase in western blot signal, the PrP detected in the initially reactive samples of this study do not have the same molecular characteristics as previously described bovine PrP^Sc^ and are therefore not concentrated by the SAF protocol (**Fig. 1**).

### Bacterial Isolation

After plating the sample homogenates, bacterial colonies were enumerated and described. The trends in bacterial populations associated with the different samples were analogous on all 3 agar types so all plate counts were averaged for each individual sample. The IR samples had high viable aerobic bacterial load but these counts were comparable to those of similar quality BSE negative samples. Surprisingly, some IR samples which appeared to be in fair condition had bacterial colony counts comparable to severely autolysed samples (**Fig. 3**). It is possible that these tissue samples contained bacterial populations at the top of the log phase of their growth curve. One or 2 more days in optimal bacterial growth conditions could have resulted in significantly more degraded tissue as the maximum microbial population was reached and their focus switched from replication to nutrient acquisition. This supports the idea that bacteria, even in IR samples which are moderately autolysed, are present in large numbers and could be involved in causing the initially reactive results.

The bacterial colonies obtained from the brain samples were similar in terms of their morphology. The primary colony types were round with regular edges and had flat to slightly convex profiles. Colony diameter after growing for 24 h at 37°C was usually between 2 to 6 mm. The majority of colonies were mucoid in appearance and clear, white or pale yellow. The mucoid appearance as well as clumpy and/or adherent growth in broth are suggestive of bacteria forming an extra-cellular matrix or biofilm to protect against adverse environmental conditions.^19^ It is possible that the resulting biofilms are not only protecting the bacteria but are also inadvertently protecting proteins from the host brain during denaturation or PK digestion. This may prevent complete PrP^C^ degradation prior to immuno-detection and result in non-specific immuno-reactivity on PK dependant BSE rapid tests.

### Sequencing

Genetic material isolated from bacteria was used to determine the sequence of the 16s ribosomal RNA present in the various isolates. The consensus sequences were compared to the sequence database of the National Center of Biotechnology Information using the Basic Local Alignment Search Tool (BLAST). The majority of the microorganisms identified were environmental or bacteria normally found on or in livestock (**Table 2**). However, several opportunistic pathogens were also identified which have been associated with central nervous system infections such as meningitis and encephalitis.^20-23^ It is possible that these bacteria were present in the brain ante-mortem. Due to the criteria of the Canadian BSE surveillance program, none of the animals tested are clinically normal and it is conceivable that the clinical symptoms which qualified the IR animal for the BSE surveillance program were caused by a central nervous system infection. Such an infection could result in host-pathogen interaction at a cellular level and would provoke a host immune response in the brain which could have an impact on BSE rapid test performance. The poor tissue quality of the IR samples prevented further exploration of ante-mortem changes in these samples.

**Table 2.** Bacteria identified in bovine brain samples using 16s rRNA amplification and sequencing

BSE negative sample bacteria	Initially reactive sample bacteria	16sRNA sequence accession number
Acinetobacter		gi│209972791│FJ405317.1
Bacillus licheniformis	Bacillus licheniformis	gi│375127273│JQ388689.1
Bacillus pumilus/safensis	Bacillus pumilus/safensis	gi│338843339│JF411291.1
Bacillus subtilis	Bacillus subtilis	gi│385251601│JQ695930.1
Carnobacterium sp.	Carnobacterium sp.	gi│308210746│AB593337.1
	**Clostridium sordellii**	**gi│211907983│FJ384388.1**
Empedobacter brevis		gi│308035680│AB517707.1
Enterococcus canintestini		gi│254952542│GQ337018.1
Enterococcus faecalis	Enterococcus faecalis	gi│317016886│HQ831431.1
Enterococcus gallinarum/casseliflavus	Enterococcus gallinarum/casseliflavus	gi│385770228│JQ805718.1
Enterococcus pseudoavium/viikkiensis	Enterococcus pseudoavium/viikkiensis	gi│359805254│AB681189.1
	**Escherichia coli**	**gi│75863990│DQ182324.1**
	**Klebsiella oxytoca**	**gi│359805791│AB681870.1**
	**Lactococcus garvieae**	**gi│237512217│FJ915634.1**
Kurthia gibsonii		gi│350577941│JN409471.1
Macrococcus bovicus		gi│343206336│NR_044928.1
Macrococcus caseolyticus	Macrococcus caseolyticus	gi│210160958│FJ263452.1
	**Pasteurella multocida**	**gi│356596244│CP003022.1**
	**Proteus penneri**	**gi│330687206│JF775423.1**
	**Shigella sonnei**	**gi│374269796│HE616528.1**
Staphylococcus epidermis/capitis/caprae	Staphylococcus epidermis/capitis/caprae	gi│347309345│JF775575.1
Staphylococcus equorum	Staphylococcus equorum	gi│342359675│JN230520.1
Staphylococcus sciuri	Staphylococcus sciuri	gi│375268447│AB697711.1
Staphylococcus succinus	Staphylococcus succinus	gi│66775052│DQ006831.1
Staphylococcus vitulinus	Staphylococcus vitulinus	gi│219856851│NR_024670.1
Streptococcus equinus	Streptococcus equinus	gi│332149382│AB563264.1
Streptococcus uberis		gi│343200133│NR_040820.1
Vagococcus carniphilus/teuberi/penaei		gi│310751478│HQ407276.1
Vagococcus fluvialis + Enterococcus sp.	Vagococcus fluvialis	gi│330895940│JF690757.1

Identification of the bacteria present in BSE negative samples and initially reactive samples. The bacteria in bold were those only found in initially reactive samples. These isolated bacteria were mixed into or cultured with known BSE negative bovine brain in an attempt to create reactivity on the immuno-chromatographic and western blot BSE rapid surveillance tests.

**FIGURE 4.**
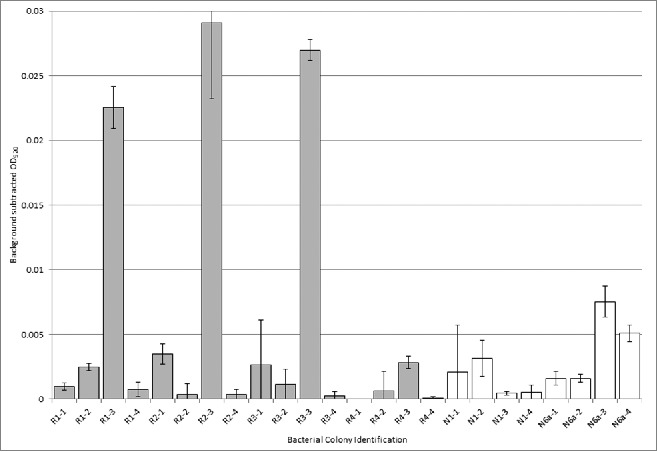
Biofilm assay optical densities for bacteria isolated from initially reactive and negative samples. One bacterial isolate from each of the first 3 initially reactive samples formed significantly more biofilm in the 24 hour growth period as represented by the increased average OD of more than 4 times greater (R1-3, R2-3 and R3-3) than bacterial isolates from negative samples (N1-1 to N1-4 and N6a-1 to N6a-4). None of the bacterial isolates tested from initially reactive sample 4 (R4-1 to R4-4) produced significantly more biofilm than the negative sample bacteria.

Comparing the bacteria found in BSE negative samples to those isolated from initially reactive samples, a total of 7 isolates were characteristic to only the reactive samples. These were *Clostridium sordellii*, *Escherichia coli*, *Klebsiella oxytoca*, *Lactococcus garvieae*, *Pasteurella multocida*, *Proteus penneri* and *Shigella sonnei*. E. coli was the only bacterium that was isolated from more than half of the initially reactive samples (3/4) while L. garvieae and P. penneri were both isolated from 2 of the 4 initially reactive samples. All of the other isolates were found in 1 out of the 4 samples. When searching the NCBI-PubMed database with individual isolate names and the word “biofilm”, *C. sordellii*, *L. garvieae* and *P. multocida* do not generate multiple article hits. The other 4 isolates (*E. coli*, *K. oxytoca, P. penneri* and *S. sonnei)* do have multiple references when searching “biofilm” and the isolates name suggesting that these microbes are biofilm formers. Results from the biofilm assay demonstrated the presence of significant biofilm forming bacteria in 3 out of the 4 initially reactive samples (**Fig. 4**). When these bacterial isolates were sequenced, 2 of the 3 were identified as stains of *E. coli* and the other was *Macrococcus caseolyticus*. *M. caseolyticus* was also found in negative sample 6 (colony ID: N6a-3), but this particular strain did not generate a biofilm when tested in our biofilm assay conditions.

To determine if growth and/or presence of any of the 7 individual bacteria only found in the initially reactive samples could cause rapid test reactivity, the isolates were inoculated into sterile BSE negative brain homogenate. All of the bacteria inoculated did grow and replicate in the brain homogenate to varying degrees (5 fold to 200 fold increase in CFU/mL). Despite successfully growing in the sterile brain homogenate, no single isolate consistently caused reactive results on any of the rapid surveillance tests for BSE. Should a single bacterial species be capable of causing BSE negative brain to test initially reactive it may require a longer incubation time and/or different growth conditions.

### Gram Staining of Tissues

Gram stained sections were read to evaluate total bacterial contamination associated with the IR, BSE negative and BSE positive brain tissues. As expected, autolysed sample sections contained the highest numbers of bacteria, regardless of their BSE test results (**Fig. 2**). The IR samples that were in fair condition also had a high number of total bacteria associated with the tissue, which corresponded well with aerobic culturing results. BSE positive/negative samples of poor and fair tissue quality had gram stained bacteria at similar levels to the IR samples of equivalent tissue quality. No specific patterns were noted in terms of bacterial type or morphology in IR samples and it does not appear that these samples contain a higher number of total bacteria.

## DISCUSSION

The Canadian BSE surveillance program targets dead, down, diseased and/or distressed cattle over 30 months of age. These samples are occasionally challenging for rapid surveillance tests to provide a conclusive stand-alone test result. Contaminants associated with the sample introduced ante mortem, post mortem or even during sampling have the potential to impact critical steps of the BSE rapid test methods. If steps are disrupted, tests can give inconclusive or initially reactive results and require additional time, money and testing to confirm their true status. The IR samples included in this study have been tested on the different BSE test platforms available to the Canadian and OIE BSE Reference Laboratory and they do not contain detectable PrP^Sc^ from the characterized types of BSE. This conclusion was based on the cumulative rapid test results (reactive only with PK based test platforms), no significant increase in PrP^Sc^ signal after SAF purification and the absence of immunohistochemistry reaction consistent with BSE positive brain of similar quality. However, a bioassay evaluation would be the most decisive way to determine if these IR samples truly do contain disease-causing prions.

In Europe, samples with similar reactivity on western blot have been inoculated into bovine transgenic mice resulting in some of the mice displaying clinical signs of neurologic disease.^11^ Consistent with the IR samples in our study, the samples injected into mice were of poor quality, autolytic and likely contained high levels of bacterial contamination. While these results are interesting and this is one of the best approaches to determine the true disease status of the samples in question, the outcome of this study must be interpreted cautiously. When the central nervous system is challenged with bacterial lipopolysaccharide (LPS), glial cell activation can result in the death of neurons via glutamate related excitotoxicity.^24^ Studies have linked PrP^C^ to NMDA receptor (a sub type of glutamate receptor) regulation and have shown that a loss of functional PrP^C^ results in an increased potential for glutamate related excitotoxicity.^25,26^ If the loss of functional PrP^C^ is a primary cause of prion disease neurodegeneration, the pathology and clinical display of a prion disease infected mouse could be very similar to that of mice injected with bacterially contaminated, prion disease negative, brain homogenate. Furthermore, if the initially reactive results on rapid tests are indicative of a new type of prion disease these same low level, abnormally reactive test results should also be detected in good quality samples. With no evidence available that good quality samples have been found which generate rapid test results similar to the samples in this and other studies, it would seem that the cause of IR samples is linked to autolysis and likely the associated high levels of bacteria.

A recent publication discusses the potential of a new strain of BSE with a similar blot profile to that seen in the Canadian IR samples.^10^ Intra-cerebral transgenic mice and cattle inoculation studies are underway for these samples; and mouse bioassay results should be available shortly. The cattle experiments have recently been terminated with no clinical signs evident but extensive testing of samples collected from these animals is currently underway (S. Czub, personal communication). Intra-cerebral inoculation of cattle is the most sensitive and relevant bioassay for BSE and should provide a definitive answer on the prion disease status of these IR samples.

With similar tissue conditions; including high numbers of viable bacteria and some degree of autolysis; shared between the Canadian IR and IR samples from other countries, it seems logical that bacteria associated with decomposition are a critical factor. Unfortunately, neither culturing nor quantification of bacteria in the IR and BSE negative samples showed a clear difference and it is unlikely the total amount of bacteria is the lone factor causing the rapid test reactivity. It is possible that a specific component, type or combination of bacteria might be required. We did show that bacteria isolated from 3 of the 4 initially reactive samples form robust biofilms. Biofilm formation is associated with increased antibiotic and environmental stress resistance by forming physical barriers and creating protective micro-environments enhancing bacterial subsistence.^12,20^ If detergents and proteases are unable to access or to function properly because of biofilm impermeability, sub optimal micro-environments or some other conformational changes to the protein, PrP^C^ may not be fully degraded resulting in a persistent signal on certain immunoassay platforms.

Sequencing of the bacterial colonies isolated from the IR BSE samples did not result in the identification of a specific bacteria or family of bacteria that cause BSE negative brain homogenate to test consistently reactive on rapid tests. With several of the bacteria identified as opportunistic pathogens of the central nervous system, an infection could result in changes to the brain tissue submitted for BSE testing. Infection can result in changes to normal cell populations, an increase in tissue cytokines as well as direct pathogen-host cell interactions. It is interesting to note that the *E. coli* chaperonin GroEL has been shown to induce a conformational change and aggregation when mixed with recombinant PrP resulting in an increase in beta sheet content and moderate protease resistance.^27^ When mixed with ovine PrP, GroEL induces similar conformational changes and aggregation which has led to the suggestion that host protein interactions with gut flora could play a role in prion disease induction.^28^ Gram negative bacteria LPS has also been shown to cause recombinant PrP to increase in beta sheet content which is more similar to the structure of PrP^Sc^.^29^ It is possible that normal PrP^C^ in the samples sent for BSE surveillance testing are interacting with bacterial components resulting in altered conformations which are more difficult to denature and/or degrade causing low level reactivity on certain PK dependant BSE surveillance tests.

While protease digestion and subsequent immuno-detection serve well for a vast majority of BSE surveillance samples, there are some samples that pose challenges. At present, there are sufficient alternative testing methods and tools to provide confidence in determining the BSE status of complex samples. However, the possible emergence of additional BSE strains or types with different properties could make future diagnosis difficult using current test methods. The potential of this scenario makes continued research to better understand protein mis-folding diseases and their diagnosis critical to human and animal health and food safety.

## DISCLOSURE OF POTENTIAL CONFLICTS OF INTEREST

No potential conflicts of interest were disclosed.
